# Silk cryogel and electrospun scaffold characterization for bone-tendon interface applications

**DOI:** 10.3389/fbioe.2026.1685458

**Published:** 2026-03-17

**Authors:** Amritha Anup, Milenka Men, Katelyn Wasacz, Michelle Bok, Afton K. Limberg, Katherine R. Hixon

**Affiliations:** 1 Thayer School of Engineering, Dartmouth College, Hanover, NH, United States; 2 Geisel School of Medicine, Dartmouth College, Hanover, NH, United States

**Keywords:** bone, cryogels, electrospinning, enthesis, interface, polyhydroxybutyrate, silk fibroin, tendon

## Abstract

**Introduction:**

Hard-to-soft tissue interfaces, such as bone-tendon or bone-ligament junctions, remain a challenge to treat. Low healing success rates stem from the complexities at the interface, creating an urgent need for better models to elucidate the properties that enable these junctions to withstand complex mechanical loads and to function as hubs for crosstalk among different cell populations.

**Methods:**

In this work, silk fibroin (SF) scaffolds fabricated via electrospinning and cryogelation were developed as an in vitro model to investigate and optimize the natural repair processes of the bone-tendon interface.

**Results:**

It was observed that electrospinning SF with polyhydroxybutyrate (PHB) as a copolymer produced scaffolds with 1-micron fiber diameters, while SF cryogels exhibited 150–200 μm pores, both of which approached native tissue dimensions. Mechanically, the electrospun scaffolds had an elastic modulus of approximately 50 MPa, compared to 0.3–0.5 MPa for the cryogels. FTIR analysis confirmed the successful combination of PHB and SF in the electrospinning process, as well as characteristic amide peaks suggesting β-sheet formation, and a degradation study provided insight to scaffold stability with time. A live dead assay confirmed cell viability with time. Cells aligned along electrospun fibers and clustered within cryogel pores from day 4 to day 12. When combined, the electrospun scaffolds and cryogels supported tendon and bone cell infiltration at days 4 and 8.

**Discussion:**

These results demonstrate that a multi-technology, multi- material tissue engineering strategy enables the creation of tunable, heterogeneous scaffolds for modeling the bone-tendon interface.

## 1 Introduction

Musculoskeletal injuries involving hard-soft tissue interfaces remain one of the most pressing challenges in orthopaedic repair. These interfaces are impacted in many musculoskeletal injuries, affecting approximately 32 million people in the United States each year (Ellis et al., 2022). Among these, injuries to the bone-tendon interface—or enthesis—are especially difficult to treat, as they involve two distinct tissues with dramatically different biological, mechanical, and structural properties. The enthesis is commonly affected in rotator cuff tears, Achilles tendon ruptures, and flexor tendon injuries, and its regeneration is essential to restoring functional load bearing across joints (Pugliese et al., 2024). However, the interface rarely regenerates with its native complexity following injury, and surgical repairs are often unsuccessful over time. In the United States alone, over 300,000 surgical procedures are performed annually to correct soft tissue degeneration at shoulder, foot, and ankle joints, contributing to over $30 billion in annual healthcare costs (Pugliese et al., 2024). Alarmingly, even after a technically successful surgical repair, the retear rate for rotator cuff injuries ranges from 13% to 94%, depending on the technique, rehabilitation protocol, and patient-specific factors (Mandaleson, 2021). These poor outcomes are typically attributed to mechanical mismatch (Bousso et al., 2024), inadequate integration at the healing interface, and the formation of fibrotic scar tissue, which lacks the biomechanical fidelity of the native enthesis (Pugliese et al., 2024; Apostolakos et al., 2014).

The bone-tendon enthesis is a highly specialized region that connects soft collagenous tendon to mineralized bone through a continuous but complex transition. This region spans only a few hundred microns in length, but contains distinct zones with varying cellular phenotypes, extracellular matrix (ECM) composition, and degrees of mineralization. Fibrocartilaginous entheses, which are the most common in the human body and frequently injured, are composed of four histologically distinct zones: (i) aligned, collagen-rich tendon; (ii) unmineralized fibrocartilage; (iii) mineralized fibrocartilage; and (iv) bone (Apostolakos et al., 2014; Leong et al., 2020). As collagen fibers from the tendon insert into bone, they pass through this gradient of increasing mineral content and decreasing fiber alignment. This gradual transition is critical for mechanical load transfer and reduces stress concentrations that would otherwise result in tissue failure. Replicating this continuous yet heterogeneous structure is a central challenge in regenerative strategies (Pugliese et al., 2024; Bousso et al., 2024; Apostolakos et al., 2014). Despite the complexity of the enthesis, current clinical approaches focus primarily on mechanically reattaching tendon to bone with sutures or anchors, often ignoring the biological and architectural gradient that is essential to long-term function (LaCroix et al., 1985).

These limitations underscore the need for tissue engineering (TE) approaches that not only restore tissue continuity but also recapitulate the spatial heterogeneity of the native bone-tendon insertion. TE offers a promising solution by combining scaffolds, cells, and bioactive factors to guide regeneration across multiple tissue types (Langer and Vacanti, 1993). Traditionally, the TE field has focused on developing single-tissue scaffolds, typically optimized for either bone or soft tissue regeneration. However, such approaches fall short when addressing multi-tissue interfaces like the enthesis. Therefore, recent advances in TE—particularly in scaffold design and fabrication—have enabled the development of composite or multilayered scaffolds that can better mimic native tissue transitions (Vesvoranan et al., 2022). Many studies utilize synthetic materials, such as poly(lactic-co-glycolic acid) (PLGA) or polycaprolactone (PCL), due to their tunable mechanical properties and widespread availability (Micalizzi et al., 2023; Ivanov et al., 2014). More recently, there has been growing interest in fabricating scaffolds from natural materials, and several studies have explored polymers such as gelatin (Olevsky et al., 2023), collagen (Qian et al., 2019; Soo et al., 2014), and silk fibroin (Chen et al., 2021). In addition to incorporating different materials, these scaffolds can be fabricated using a variety of processing techniques. Importantly, combining multiple fabrication methods within a single construct allows for spatial tuning of properties such as porosity, swelling behavior, mineral content, and stiffness across the scaffold. Common fabrication techniques for bone and tendon include cryogelation and electrospinning, respectively, where each method offers unique benefits depending on the target tissue. Cryogels form macroporous, sponge-like scaffolds with interconnected pores that are advantageous for bone due to their mechanical robustness, vascularization support, and resemblance to the mineralized trabecular matrix (Hixon et al., 2017a). Electrospinning, in contrast, creates nanoscale fibrous mats that replicate the aligned, dense collagen architecture of tendon, promoting cell alignment and tenogenic differentiation (Xue et al., 2019).

To fabricate a multi-component scaffold capable of supporting both bone and tendon regeneration, materials must be carefully selected to ensure compatibility with each fabrication technique, while also maintaining bioactivity, biocompatibility, and mechanical integrity. One such material is silk fibroin (SF), a natural protein derived from *Bombyx mori* silkworms that is FDA-approved for multiple clinical applications (Wani et al., 2022; Rockwood et al., 2011). SF has been used in sutures, wound healing, and drug delivery systems due to its tunable degradation rate, low immunogenicity, biocompatibility, and favorable mechanical strength (Wani et al., 2022). The hydrophobic β-sheet domains in SF provide structural integrity, while hydrophilic regions support solubility and processing. The balance of these domains can be manipulated through physical or chemical triggers (e.g., sonication, pH, or temperature) to yield TE scaffolds with desirable properties (Rockwood et al., 2011; Samal et al., 2013). Importantly, SF is compatible with both cryogelation and electrospinning, enabling the development of dual-fabrication scaffolds that integrate bone-like and tendon-like features. For cryogelation, aqueous SF is exposed to freezing conditions and sonication to induce gelation via β-sheet formation. The resulting cryogel exhibits high porosity and mechanical resilience—properties essential for bone tissue support (Hixon et al., 2017a; Wani et al., 2022). For electrospinning, SF is often blended with a co-polymer to stabilize the spinning process and improve fiber formation. In this study, polyhydroxybutyrate (PHB), a biodegradable natural polymer produced by microorganisms, was selected as a co-polymer. PHB enhances SF electrospinnability, allowing for the fabrication of aligned, fibrous mats that mimic the parallel collagen fiber arrangement seen in tendon (Karahali̇Loğlu, 2017).

Here, we present a proof-of-concept composite scaffold that combines SF-PHB electrospun mats and SF cryogels to model the mechanical, structural, and biochemical gradients found at the bone-tendon enthesis. To the best of our knowledge, this is the first study to combine cryogelation and electrospinning to address bone-tendon interface engineering using exclusively natural polymers We first characterize each component individually by evaluating their morphology (i.e., fiber diameter, fiber alignment, pore size), mechanical behavior (i.e., tensile and compressive properties), and cytocompatibility using relevant cell types. We then integrate both scaffold components into a composite construct and assess the feasibility of engineering a seamless hard-soft bone-tendon interface. Our goal is to identify critical design parameters that influence scaffold performance and cellular response in the context of a graded, spatially organized interface. This work contributes to a growing body of research focused on functional tissue interface engineering and provides a platform for future *in vivo* studies investigating regeneration at hard-soft tissue junctions.

## 2 Methods

### 2.1 Silk fibroin (SF) extraction process

The process of silk fibroin (SF) extraction is well-established and previously described in literature (Rockwood et al., 2011). Briefly, day one consisted of cutting cocoons (Treenway Silks) into dime size pieces (four to five pieces per cocoon) and removing the worm. Five grams of cocoon were then boiled in 0.02 M sodium carbonate (Na_2_CO_3_; 223,530, Sigma) for 30 min, which removed the sericin coating on the SF. Next, cocoons were rinsed three times in deionized (DI) water at room temperature, 20 minutes per rinse. Once washing was complete, excess water was squeezed out and fibers were left to dry overnight. Day two began by adding 9.3M of lithium bromide (LiBr; 213,225, Sigma) on top of the SF fibers and incubating at 60 °C for 4 hours. This converted SF to an aqueous solution form that was then transferred to a dialysis cassette (66,333, Thermo Scientific Slide-A-Lyzer, 3.5K MWCO) using a 20 mL syringe (18,762, BD) and an 18 G needle (305,195, BD). Dialysis in DI water was performed for 48 h total, with the water changed at timepoints one, four, 5 hours (day two), the next morning and evening (day three), and ending on the morning of day four (for a total of six water changes). The SF was then removed from the cassette and centrifuged twice at a speed of 12,700g to remove any remaining debris.

To determine the concentration of the SF solution, three weigh boats were each filled with 0.5 mL of the SF solution. The filled weigh boats were then placed in an oven at 60 °C for complete drying, leaving behind a film of pure SF. This was then massed and, by taking the difference in masses divided by 0.5 mL and multiplying by 100, the result is the total % SF in solution. Note that this SF stock solution can be stored at 4 °C for up to two to 4 weeks. This stock solution can also be lyophilized and stored for more than a year in a desiccator at room temperature (Rockwood et al., 2011).

### 2.2 Fabrication of electrospun mats

Electrospinning solution was created by combining different ratios of PHB (BU39-GL-000111, Goodfellow) to lyophilized SF in 12 mL of hexafluoro-2-propanol (HFIP; 3409, Oakwood Chemical) solvent. Total polymer concentrations were standardized at 4, 6, and 8% (w/v) with each polymer concentration divided into groups of PHB:SF ratios (0:100, 25:75, 50:50, 75:25, 100:0). For example, 0.18 g of PHB and 0.54 g of SF were combined with 12 mL of HFIP to formulate a 6% polymer sample with a 25:75 ratio of PHB:SF. An overview of solution composition is summarized in Table 1. Once combined, solutions were placed on a mechanical shaker overnight (115 RPM) to ensure the complete dissolution of PHB and/or SF in the solvent while limiting bubble formation. All solutions achieved full transparency and were immediately electrospun within 48 h.

**TABLE 1:** ES solution compositions. Column one and three both describe the total polymer used to make the solution (% of total mass and mass in grams, respectively). The specific masses of SF and PHB are also provided. ES solutions were made by mixing these pre-determined amounts of polymer into 12 mL of HFIP.

Total % polymer	PHB:SF	Total polymer (g)	SF (g)	PHB (g)
4	100/0	0.48	0.00	0.48
	25/75		0.36	0.12
	50/50		0.24	0.24
	75/25		0.12	0.36
	0/100		0.48	0.00
6	100/0	0.72	0.00	0.72
	25/75		0.54	0.18
	50/50		0.36	0.36
	75/25		0.18	0.54
	0/100		0.72	0.00
8	100/0	0.96	0.00	0.96
	25/75		0.72	0.24
	50/50		0.48	0.48
	75/25		0.24	0.72
	0/100		0.96	0.00

For the electrospinning process, the PHB and/or SF solution was loaded into a 20 mL Luer-Lock syringe fitted with an 18 G needle and connected to the Spinbox Advanced Electrospinning machine (Bioinicia Fluidnatek). A positive voltage was applied to the syringe needle tip as the solution was extruded at a set rate. Ten mL of solution was spun and collected for each sample. The polymer fibers were collected on a rotating drum with an applied negative voltage, where the drum was covered in a layer of carbon-loaded polyethylene material (00631546024375, International Plastics) to ensure seamless removal of the electrospun (ES) fibrous mat. Parameters including voltage, working distance, and flow rate were varied slightly for each solution to optimize the Taylor cone, minimize beading/webbing of fibers, and adapt to the variable environmental temperature and humidity (Sarıkaya and Gümüşderelioğlu, 2021). The specific processing parameters for all scaffold variations are detailed in Supplementary Table S1. For the final 6% 50:50 scaffold, a stable jet was achieved at a flow rate of 8 mL/h, a working distance of 13 cm, and voltages adjusted to maintain a potential difference of ∼13 kV. After collecting fibers for 1 hour per solution, the ES fibrous mats were carefully removed from the drum and stored in air-tight plastic bags prior to analysis. A total of three separate ES mats were fabricated per group for analysis.

### 2.3 Fabrication of cryogels

The fabrication of SF cryogels (CGs) was optimized based on previous studies published by our group (Ivanov et al., 2014; Hixon et al., 2017a; Xue et al., 2019). For each sample, 3 mL syringes with caps secured onto the luer-lock tips were pre-frozen via submersion through foam floating in a −20 °C methanol bath for at least 1 h prior to CG fabrication. Once syringes were precooled, the SF stock solution was retrieved from the 4 °C fridge, along with the pre-cooled syringes from the −20 °C freezer, and placed into an ice bucket to keep chilled. Next, 2 mL of SF solution was pipetted into the pre-cooled syringe and placed in a small ice bath, where the sonication probe (FB-120, ThermoFisher) was inserted such that the tip was ∼2 cm within the SF solution. Sonication settings were 80% and the sonication time was set to 30 s, where pulse settings were adjusted between no pulse (NP), 1 s on/off (Ellis et al., 2022), 2 s on/off (Pugliese et al., 2024), and 3 s on/off (Mandaleson, 2021), depending on the sample. The maximum power of this probe is 120 W, therefore at 80% amplitude, the power delivered is 96 W. A foam sample holder was used to stabilize the syringe in the beaker while sonicating. Immediately following sonication, the syringe was removed from the ice and gently tapped to disperse air bubbles, which created an even surface at the top of the syringe. Then, the plunger was affixed and the sealed syringe was quickly placed in the −20 °C freezer methanol bath for 24 h. Following 10 minutes of thawing at room temperature, the CG was removed and prepared for long-term storage via lyophilization. For lyophilization, CG samples were placed in the −80 °C freezer for 1 hour and then transferred to a lyophilizer (FreezeZone Freeze Dryer, Labconco) for a minimum of 24 h. Following lyophilization, CGs were cut into 10 mm segments and stored in a desiccator until use. Note that for each sample type, three to five syringes (1180300777, McKesson) of CG were fabricated, and individual samples were randomly assigned to analysis groups.

### 2.4 Anisotropic analysis

To assess fiber alignment, analysis was performed on 1,000x magnified SEM images for each sample to quantify fiber alignment using the FibrilTool ImageJ plug-in (Rockwood et al., 2011). Each image was divided into quadrant ROIs in ImageJ using the polygon tool and then analyzed using FibrilTool to obtain fractional anisotropy (FA) values, ranging from 0 to 1, with 0 indicating no alignment and one indicating 100% parallel alignment of fibers. The anisotropy values from the four ROIs were averaged to obtain the average anisotropy of each sample. For each polymer ratio-concentration blend, the average anisotropy values of all three samples were then averaged to obtain the average polymer blend anisotropic value. Note that following anisotropy analysis and based on the results, only groups 75:25, 50:50, and 25:75 were chosen for continued analysis to more closely investigate scaffold properties of polymer blends.

### 2.5 Electrospun scaffold fiber diameter

Scanning electron microscopy (SEM; VEGA3 TESCAN) images were processed using ImageJ (NIH) software to characterize the fiber diameter of each sample. The samples (N = 3/group) were mounted on an aluminum stub and sputter coated (HUMMER 6.2) for 240 s in gold at 15 mA under the pulse setting to avoid overheating before being imaged. Images were taken at 500x, 1,000x, 3000x, and 5,000x magnifications. Briefly, each 3000x magnified image was uploaded into ImageJ and the line tool was used to scale the image using the SEM scale bar according to pixels per known micrometer. The image was then split into four quadrants as regions of interest (ROI) in ImageJ. Using the “Measure” function, 15 individual fibers were selected and analyzed in each quadrant for a total of 60 fiber diameters, based on previous studies (Hixon et al., 2017b). These diameter measurements were averaged to obtain the average fiber diameter of each sample. For each polymer ratio-concentration blend, the average fiber diameters of all three samples were then averaged to obtain the average polymer blend fiber diameter.

### 2.6 CG scaffolds pore diameter

SEM was used to observe the pore structure of the CGs. Like the ES scaffolds, CG samples (N = 3/group) were mounted on an aluminum stub and sputter coated (HUMMER 6.2) for 240 s in gold at 15 mA under the pulse setting to avoid overheating. SEM was then used to obtain CG images at ×100 and ×200 magnifications. ImageJ was also used to analyze the pore diameter in the CG scaffolds. First, the image was divided into quadrant ROIs in ImageJ. Then, the line tool was used to scale the image using the SEM scale bar according to pixels per known micrometer. The unit of length was adjusted to microns and the freehand selection tool was used to draw the perimeter of the pore. Feret diameter of the pore was measured in microns (µm). The measurement was repeated 15 times per ROI, for a total of 60 different pores per image. The data were saved in an Excel file and the measurements were used for statistical analysis.

### 2.7 Swelling kinetics

To evaluate the shape retention and rehydration potential of CGs, a swelling test was performed. The dry weight for all lyophilized CG scaffolds (N = 3/group) was recorded at the start of each test. Each sample was then placed in a weigh boat containing 5 mL of DI water, removed, and weighed at nine different time points: 2, 4, 10, 20, 40 min and 1, 2, 4, 24 h. The average swelling ratio, considering the original dry mass of each sample, was calculated using the established equation:

$$Swelling\;Ratio=\frac{W_{H}-W_{D}}{W_{D}}$$

Here, $W_{H}$ is the hydrated weight and $W_{D}$ is the dry weight (Kathuria et al., 2009).

### 2.8 Mechanical testing

To characterize mechanical properties of the scaffolds, compression for CG/bone and tensile for ES/tendon (N = 3/group) was done. Ultimate compression testing was conducted to 50% for all CG scaffolds following hydration for 5 minutes in phosphate buffered saline (PBS; BP39920, Fisher Scientific). Compression testing was completed using an Intron 68SC-2 system (Instron) with a 100 N load cell and set parameters of a test rate of 10 mm/min, preload of 0.05 N, and preload speed of 1 mm/min. For tensile testing, ES mats were cut into 2 × 6 cm rectangles and fixed onto paper frames using Loctite and double-sided tape, following a published protocol (Maccaferri et al., 2021). Sample height was set to 10 mm and noted on the sample using two denoted spots near the center. Parameters were set to a test rate of 10 mm/min and preload of 0.05 N, and samples were strained up to 27% or failure. All data were analyzed through the Bluehill universal software (Instron). Both compressive and tensile modulus (MPa) for ES and CG scaffolds, respectively, was calculated by identifying the slope of the resulting stress-strain curve.

### 2.9 Structural insights with FTIR analysis

Fourier-transform infrared spectroscopy (FTIR, FT/IR-6200, Jasco) equipped with an attenuated total reflectance (ATR) accessory was used to analyze the chemical composition of the selected scaffolds. To prepare samples, cryogels were ground into a fine powder using a mortar and pestle; the electrospun scaffolds were analyzed directly without preprocessing due to their thin-film nature. For each sample, 32 scans were collected at a resolution of 4.0 cm^-1^.

### 2.10 Enzymatic and hydrolytic degradation assays

Four samples of each finalized scaffold formulation (6% ES and 6.5% CG) were incubated hydrolytic and enzymatic degradation studies. Hydrolytic degradation was assessed by incubation in phosphate-buffered saline (PBS), while enzymatic degradation was evaluated using protease (1 U/mL, *Streptomyces griseus*, Protease XIV, Sigma) and/or lipase (10 U/mL, *Candida rugosa*, Sigma). Samples were incubated at 37 °C for 3, 14, or 21 days. Lipase-containing conditions (lipase alone and lipase + protease) were applied only to electrospun scaffolds due to the presence of PHB, an ester-based polymer susceptible to lipase-mediated degradation. Degradation solutions were refreshed every 5 days. Mass loss was quantified by weighing the scaffolds before and after incubation and calculating the remaining mass% as follows:

$$Mass\;\%\;Initial=\frac{M_{f}}{M_{i}}*100$$

The scaffolds were then freeze-dried and imaged using SEM to assess changes in surface topography.

### 2.11 Combined ES and CG scaffold fabrication

Next, the combined scaffold structure which forms the bone-tendon interface was fabricated. The optimal ES and CG scaffolds were determined to be 6% 50:50 blend of PHB:SF and 6.5% three to three pulse, respectively, due to the desired tissue qualities described in the introduction and results sections. Following the previously mentioned CG fabrication, combined scaffolds were created by directly electrospinning 6% 50:50 PHB:SF solution onto 6% 3–3 CGs that were physically attached via tape to carbon-loaded polyethylene material on the collector drum. The same parameters were used for the cryogelation and electrospinning process, and samples were removed carefully by first detaching the polyethylene material, cutting the ES portion and carefully removing the CG from the tape.

### 2.12 Bone-tendon cell culture

To quantify the relevance of ES and CG scaffolds for tendon, bone, and the bone-tendon interface, 2 cell types were chosen: tenocytes extracted from mouse Achilles tendon and differentiated, mouse-derived, pre-osteoblastic MC3T3-E1 Subclone four cell line (ATCC).

#### 2.12.1 Mouse tenocyte extraction

Mouse tenocytes were extracted by digesting Achilles tendons harvested from both the left and right hindlimbs of healthy adult C57BL/6 mice (aged 12–16 weeks) of both sexes. Tendons were collected immediately following euthanasia conducted under an institutionally approved animal use protocol. All tendons were harvested immediately following sacrifice by creating a skin incision slightly lateral to the Achilles tendon, exposing and isolating the tendon by passing a pair of sterile dissecting scissors underneath it, and then cutting at first the proximal (myotendinous junction) and then distal (enthesis) end (Dudakovic et al., 2024). Dissected tendons were immediately placed in a solution of αMEM (12571063, Thermo Fisher Scientific) and 2 mg/mL collagenase type 1 (9001-12–1, Worthington Biomedical Corporation). The tendon-containing solution was left in a warm water bath for 1 hour and vortexed every 10 min. After this incubation period, the tendon containing solution was filtered using a 70 μM filter to remove any remaining tendon pieces. The solution was then spun down at 125 g for 7 min to obtain a cell pellet, and this was then resuspended in tenocyte media composed of αMEM, 1% pen strep, 1% glutaMAX, and 10% fetal bovine serum (FBS). The cell solution was then diluted to one million cells per mL in freezing media mixture containing 70% tenocyte media, 20% FBS, and 10% DMSO, and frozen in liquid nitrogen until use. A frozen vial of cells was cultured by thawing and subsequent incubation at 37 °C and 5% CO2 at a concentration of ∼2 million cells in a T-75 flask.

#### 2.12.2 Differentiation of MC3T3-E1 subclone 4 pre-osteoblasts

The second cell type used for *in vitro* studies were differentiated cells of pre-osteoblast lineage. Differentiation of the MC3T3-E1 Subclone four cell line was conducted by culturing cells in differentiation media as per published protocols (Quarles et al., 1992; Yoon et al., 2025; Semicheva et al., 2024; Wang et al., 1999). The differentiation media for the pre-osteoblastic cells used in this study consisted of αMEM, 10% FBS, 1% penicillin-streptomycin (P/S), 100 μg/mL ascorbic acid, and 3 mM of B-glycerol phosphate. The cells were cultured in this media for 2 weeks before seeding onto the scaffolds. By this time, cells had reached ∼75% confluency and passage three to five.

#### 2.12.3 Scaffold and control cell seeding

Prior to cell culture, all scaffolds (ES, CG, combined) and coverslips were subjected to a 70% ethanol soak for 1 hour followed by three PBS rinses for 20 min each. A 10 mm biopsy punch was used to cut uniform pieces of the ES scaffolds, which were then seeded with 30,000 murine tenocytes. CG scaffolds were cut to 5 mm lengths (diameter of 8 mm) and seeded with 30,000 osteoblastic cells. Coverslips coated in PLL (NC0818511, Neuvitro), used as non-scaffold controls, were also seeded with either 30,000 tenocytes or osteoblasts. To seed the combined ES and CG scaffolds, 30,000 tenocytes were first seeded on the ES portion, followed by 20,000 osteoblastic cells on the CG, and combined scaffold media consisted of a 3:2 ratio of tenocyte:osteoblast media. A coverslip was seeded with tenocytes and osteoblasts on separate sides of the coverslip as the control for the combined scaffold. All scaffolds were cultured for 4, 8, or 12 days to assess adhesion and spreading. Media was changed every 2–3 days for all culture wells.

#### 2.12.4 Confocal microscopy

At the defined timepoints, scaffolds (and coverslip controls) were fixed in 4% Paraformaldehyde (101,176-014, Fisher Scientific) overnight, then washed in PBS three times for at least 5 minutes each. Anti-vinculin (F7053, Sigma) was diluted to 1:40 and 250 µL was administered dropwise to each scaffold, followed by incubation overnight at 4 °C covered in aluminum foil. Next, scaffolds were incubated in a 1:200 dilution of Rhodamine phalloidin-TRITC (HB8621, HelloBio) in a 50:50 solution of blocking buffer and 0.1% Triton X-100 for 2 h. Lastly, scaffolds were incubated with 1:1,000 4′,6-diamindino-2-phenylindole (DAPI; D1306, ThermoFisher) in PBS for 10 min. Confocal images were taken on a Spinning Disk Confocal Microscope (Andor).

#### 2.12.5 Live/Dead staining

Live and dead cells were assessed visually using fluorescence-based viability staining days 4, 6, and eight post seeding. Briefly, the CytoCalcein™ Green solution was utilized to label viable (live) cells green, while the Propidium Iodide was used to label non-viable (dead) cells red, according to the manufacturer’s protocol (AAT Bioquest; Pleasanton, CA). After incubation with the staining solution for 60 min, samples were imaged at ×4 magnification using an EVOS M5000 fluorescence microscope ThermoFisher, Waltham, MA) and qualitatively assessed for cell death.

### 2.13 Statistical analysis

All statistical analyses and graphical figures were created using GraphPad Prism version 10.3.1 (GraphPad Software). Two-way ANOVA analysis was done for quantification of anisotropy ratio, fiber diameter, and elastic modulus, and one-way ANOVA was used to quantify pore diameters of CGs, both with Tukey *post hoc* when comparing multiple groups. A p-value of <0.05 was considered significant.

## 3 Results

### 3.1 ES scaffold morphology

#### 3.1.1 Anisotropy analysis

**FIGURE 1:**
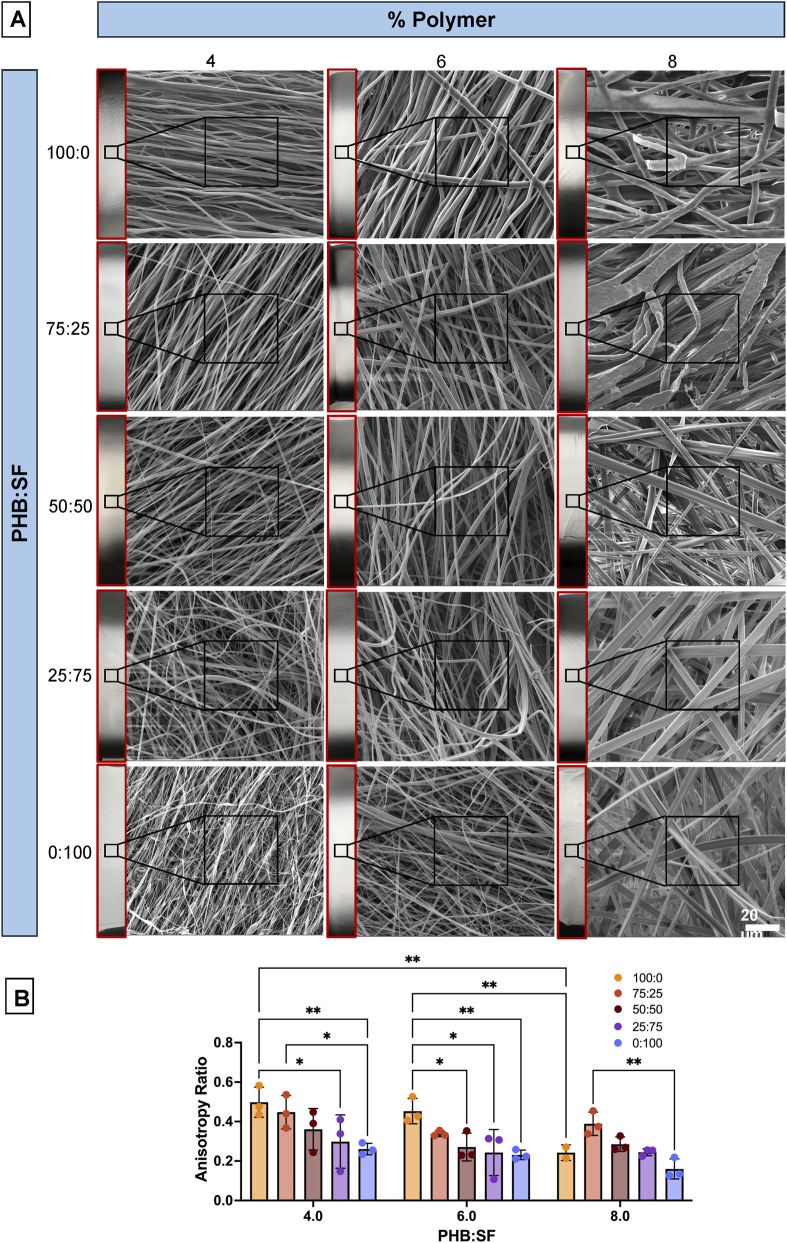
Morphological characterization of ES scaffolds. **(A)** Boxed in red are macro-scale insets of electrospun fibers for the 100:0, 50:50, and 0:100 samples. SEM images (3000x magnification) of all electrospun scaffold variations demonstrates fiber orientation. **(B)** Anisotropy ratio quantified using ImageJ for all sample variations; * = p < 0.05, ** = p < 0.005, *** = p < 0.0005. N = 3 for all groups except for 100:0, as the high concentration in 8% PHB only made it difficult to spin, so only two samples with distinct fibers were able to be created for demonstration.

Visual analysis of ES scaffold SEM images, as well as anisotropy assessment, was completed prior to mechanical analysis. From the SEM summary depicted in Figure 1A, visual assessment suggested that ES mats made with higher polymer content and more PHB resulted in larger fibers. Anisotropy values were calculated for each mat and results suggested that as SF content increased, alignment decreased (0.0001) and as % polymer increased, alignment decreased (0.0014). Further, multiple comparisons analysis resulted in significant differences between several samples as shown in Figure 1B (p < 0.05). Note that an increased variation in anisotropy is most closely related to changes in PHB:SF ratio as opposed to % polymer; higher PHB content resulted in a higher and therefore more favorable anisotropy ratio. Although 100% PHB samples exhibited more favorable anisotropy values, these values were not significantly improved from 75:25 PHB:SF samples across all % polymer samples (p = 0.05).

Additionally, 100:0 (100% PHB) mats exhibited an extremely brittle structure, making these scaffolds more difficult to detach from the collector. Figure 1A shows macro-scale insets to provide visual evidence for differences in the spread of fibers on the mandrel for different polymer ratios and presence of aberrations. For 100% PHB (100:0) samples, there was a large spread of fibers, branching, and physical aberrations (especially visible in 4% and 8% 0:100, Figure 1A). Further, 0:100 samples (100% SF) were not ideal due to the presence of beading (especially at 4% polymer concentration, indicating the polymer concentration was too low), inconsistent fiber diameters, and very low anisotropy (Figures 1A,B, 4% 0:100 image). Therefore, 100:0 and 0:100 samples were eliminated and the remainder of the study focused on all 75:25, 50:50, and 25:75 samples to optimize the PHB-SF blend while simultaneously capturing the favorable properties of both polymers in an ES scaffold.

#### 3.1.2 Fiber diameter analysis

**FIGURE 2:**
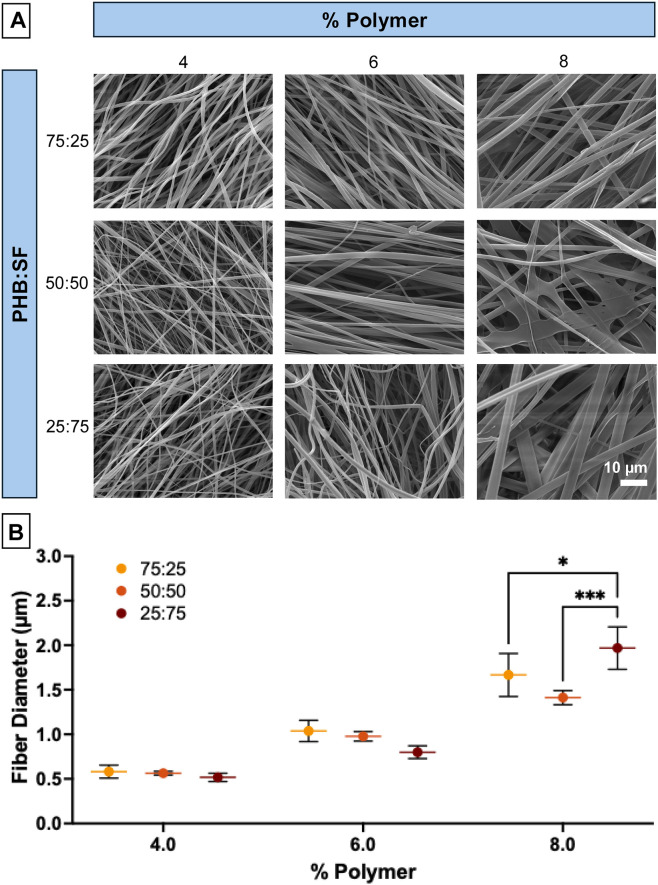
Further quantification of morphological characteristics of select ES scaffolds. **(A)** SEM zooms (x5,000) of 75:25, 50:50, and 25:75 PHB:SF blended sample variations. **(B)** Fiber diameter measured using ImageJ (n = 3). All samples with the same PHB:SF ratio and differing only in % polymer (e.g., 4% 50:50% vs. 6% 50:50) were significant (p < 0.05); these significances are not shown on the graph to maintain clarity.

SEMs at a higher magnification reported in Figure 2A depicted finer features of the 75:25, 50:50, and 25:75 samples. Fiber diameter increased with increased polymer concentration (4%–8%), and some webbing (or thickening of fibers) can be observed in the 50:50 and 25:75 8% samples, therefore these samples were eliminated. Fiber diameters were quantified using ImageJ and observed to fall within the micron scale, confirming these mats to be microfibrous (Figure 2B). Significant differences were found between the % polymer (p < 0.0001) and the interaction between % polymer and PHB:SF ratio (p = 0.001). As the SF component in these blended scaffolds increased, fiber diameter also trended to decrease in all groups except 8%. All samples with the same PHB:SF ratio and differing only in % polymer (e.g., 4% 50:50% vs. 6% 50:50) were significant (p < 0.05); however, these significances are not shown on the graph to maintain clarity.

#### 3.1.3 Tensile testing analysis

**FIGURE 3:**
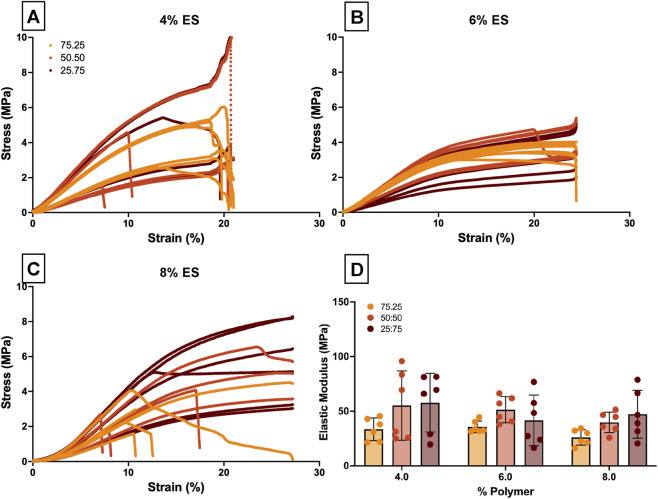
Mechanical properties of scaffolds obtained via compression testing. **(A-C)** Raw stress strain spectra for electrospun fibers (4, 6, and 8% polymer) tensile tested to failure or up to 27% strain. **(D)** Elastic moduli (mean and raw data), with results determined during tensile testing (n = 6). * = p < 0.05, *** = p < 0.0005.

After collecting stress-strain curves from tensile testing of ES scaffolds (Figures 3A–C), elastic moduli values were determined (Figure 3D). While there was a slight significance in variation with PHB:SF ratio (p = 0.0109), multiple comparisons analysis did not support any significant pairs (the lowest p-value observed was between 75:25 vs. 25:75 for 4% at p = 0.07). IQR evaluation, confirmed nooutliers. When looking at the results for each % polymer, it can be noted that as the SF content increased, there appeared to be an increased spread of the data points. This indicates greater sample fiber variability as SF content increases, which could also be visually observed in SEMs as shown in Figure 2A.

The final scaffold composition was chosen to be 6% 50:50 scaffolds due to its relatively high anisotropy, more precise and high elastic modulus values, and fiber diameter in the biologically-relevant 1-micron range (Zhang et al., 2021). Additionally, as this chosen ES scaffold contains both SF and PHB, the positive attributes of both polymers can be represented within the final scaffold; specifically, biological compatibility from SF (Wani et al., 2022) and electrospinning stability from PHB (Karahali̇Loğlu, 2017).

### 3.2 CG scaffolds morphology

#### 3.2.1 Pore diameters and porosity analysis

**FIGURE 4:**
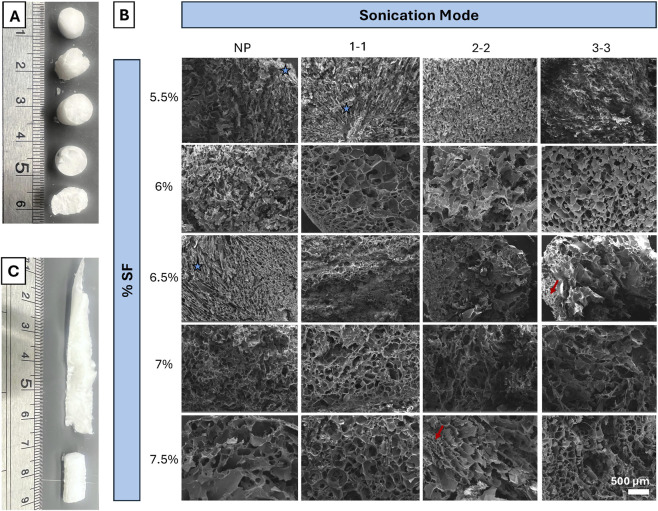
Visual morphological characterization of CG scaffolds. **(A)** Macro-scale view of cryogels, from top to bottom: 5.5%, 6%, 6.5%, 7%, 7.5%. Red arrows indicate clusters of smaller pores within larger pores. Blue stars mark instances of elongated pores. **(B)** SEM images of all CG scaffold variations, ×200 magnification, depicting pore distribution and sizes. **(C)** Representative images of 5.5% SF cryogels, one of which poorly crosslinked, resulting in a misshapen gel.

**FIGURE 5:**
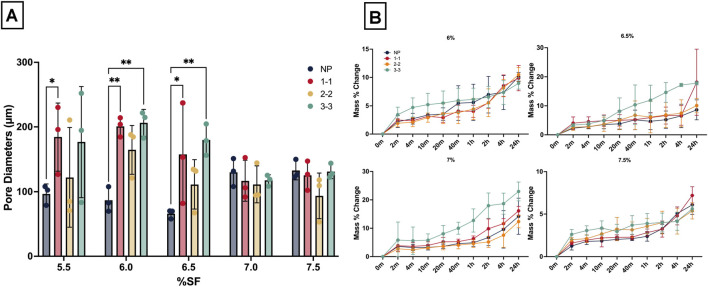
SF cryogel morphological quantification. **(A)** Pore diameters quantified via ImageJ, where each point represents the average size of 60 pores (N = 3), and **(B)** swelling test results. * = p < 0.05, ** = p < 0.005.

SF CGs were also fabricated from a variety of SF concentrations (%) and pulse settings (NP, 1-1, 2-2, 3-3). Figure 4A depicts 3–3 CGs at each % SF; it can be visually noted that the 7.5% SF CG appear slightly misshapen with a jagged cross section, which is likely due to the high polymer concentration. Figure 4B SEM images further depict the large variety of pore sizes of all the SF CGs. Interestingly, several sample types (i.e., 6.5% three to three% and 7.5% two to two, marked by red arrows) feature a set of smaller pores clustered in groups, surrounded by much larger pores. Most pores appeared ellipsoidal, there are a few instances of elongated pores (i.e., 5.5% NP and 1-1, 6.5% NP, marked by blue stars). Figure 4C depicts high variability in the 5% samples, likely due to hitting a lower threshold for polymer content for scaffold development. In Figure 5A, pore diameters were quantified for each sample type. There were a few samples that featured high variability in pore sizes, especially in 5.5% samples. As SF concentration increased to 7% and 7.5%, there were no differences in pore diameters between treatments (p > 0.05).

#### 3.2.2 Swell testing analysis

Figure 5B shows swell test results for samples from timepoints ranging from 2 minutes to 24 h. Pulsed three to three samples trended toward having the largest mass% change for most timepoints, especially earlier timepoints, although the differences were not significant (p > 0.05). However, time was a consistently significant factor for variation for all sample types (p < 0.0001). Post multiple comparisons analysis, 6.5, seven% and 7.5% had several pairs that were significantly different starting from timepoint 1 h onwards, although these were not shown on the graph for readability. Notably, 7% samples displayed the largest differences in mass% change readings among groups, with the largest change exceeding 20% for the 7% three to three samples. Note that 5.5% CG samples broke into several pieces when in contact with the PBS solution, therefore swell testing could not be completed for these samples. Therefore, these samples were eliminated from further analysis.

A summary of significance at 2, 4, and 24 h is provided in Table 2, where 7.5% samples only had one significant comparison between between 1-1 and 2-2.

**TABLE 2:** Summary of significance levels for pulse comparisons of 6.5, 7, and 7.5% SF CG during swell tests at 2, 4, and 24 h 6% CG are not included because there were no significant comparisons in this group.

Time	2 h			4 h			24 h		
% polymer	6.5	7.0	7.5	6.5	7.0	7.5	6.5	7.0	7.5
NP vs. 1–1	ns	ns	ns	ns	ns	ns	**	ns	ns
NP vs. 2–2	ns	ns	ns	ns	ns	ns	ns	ns	ns
NP vs. 3–3	ns	****	ns	**	***	ns	**	**	ns
1–1 vs. 2–2	ns	ns	ns	ns	ns	ns	*	ns	*
1–1 vs. 3–3	ns	**	ns	*	*	ns	ns	*	ns
2–2 vs. 3–3	ns	****	ns	*	****	ns	*	****	ns

#### 3.2.3 Compression testing analysis

**FIGURE 6:**
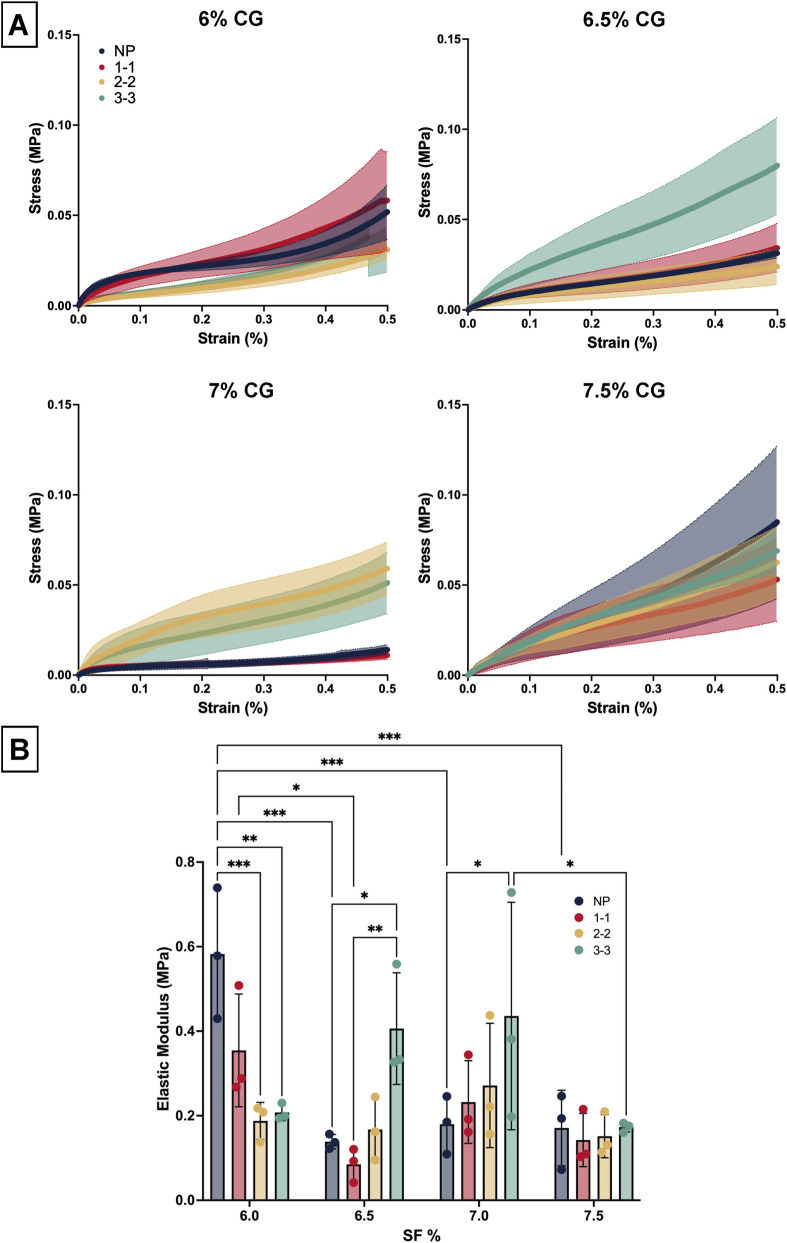
**(A)** Stress strain spectra for cryogel compression testing up to 50% strain. **(B)** SF cryogel elastic modulus extracted from compression testing stress/strain results for four variations of CG (N = 3). * = p < 0.05, ** = p < 0.005, *** = p < 0.0005.

Figure 6 summarizes the elastic modulus values of each CG from compression testing studies. The seven% and 7.5% CGs only have one pair of significantly different results (three to three comparison, p = 0.0294), therefore the elastic modulus did not significantly change when going from seven% to 7.5% SF. For 6% SF, the two to two and three to three pulsed samples had lower elastic modulus than NP (p < 0.001). For 6.5% SF, this trend was reversed, where NP and one to one samples had less elastic modulus than three to three pulse (p < 0.0295). Since 6.5% 3–3 CGs and 6% NP scaffold types had the largest elastic modulus, these sample types were the most resistant to change in strain due to force.

Integrating findings from the various CGs tested, we concluded 6.5% three to three gels had larger average pore diameters (175–200-micron range), good swelling capacity, and a relatively high elastic modulus, therefore this sample type was identified as the optimal scaffold type to use for the combined scaffold.

### 3.3 FTIR analysis

FTIR-ATR spectra of both the CG and the ES scaffolds confirmed the presence of silk fibroin in both materials, with the ES scaffolds additionally exhibiting characteristic features of PHB. Both scaffold types displayed a prominent Amide I peak at approximately 1,621 cm^-1^, consistent with the β-sheet-rich secondary structure of silk fibroin. In the electrospun scaffolds, the carbonyl stretching vibration associated with ester groups in PHB was observed as a distinct and dominant peak at 1723 cm^-1^, confirming successful incorporation of PHB into the composite fibers.

### 3.4 Enzymatic and hydrolytic degradation study

**FIGURE 7:**
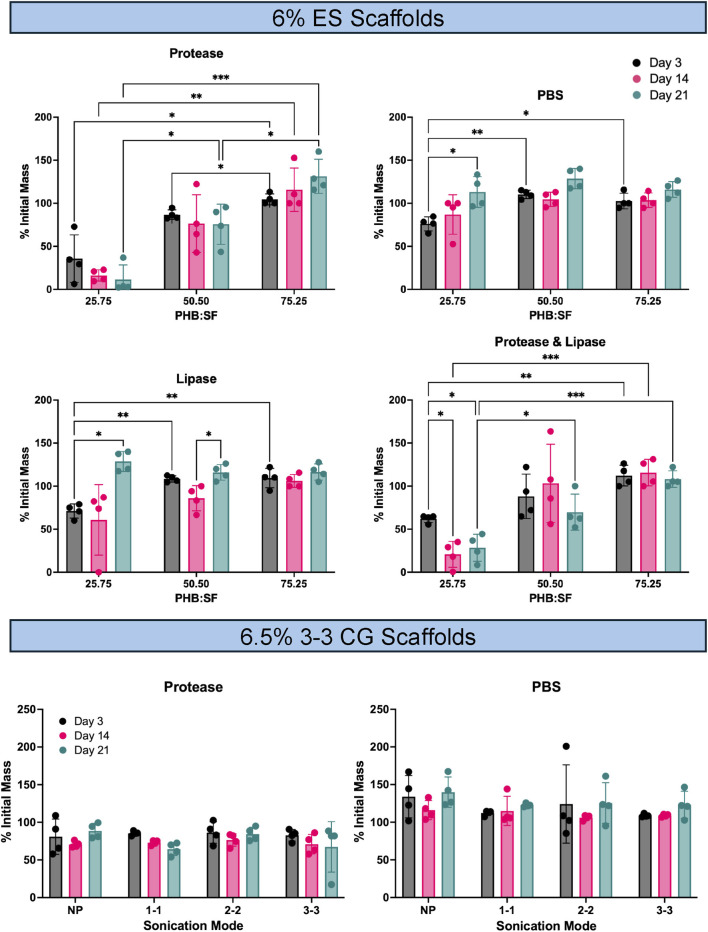
Degradation study conducted on the final chosen ES and CG samples.

Degradation behavior of the selected scaffold formulations is summarized in Figure 7. Incubation of both scaffold types in PBS resulted in minimal changes in mass across all timepoints, indicating limited hydrolytic degradation. For ES, significant differences were observed between the D3 mass measurements across treatment scaffold groups. In contrast, cryogel scaffolds exhibited no significant differences in percent initial mass under either protease or PBS incubation conditions at any timepoint. Among ES formulations, the 75:25 PHB: SF scaffolds showed the least significant changes in mass over time, with values remaining at or above 100% of the initial mass across all treatment conditions, indicating minimal mass loss and potential fluid uptake. In comparison, the 25:75 PHB:SF scaffolds exhibited the highest rates of degradation across all treatment groups, consistent with increased silk content and greater susceptibility to enzymatic and hydrolytic degradation.

### 3.5 ES and CG scaffold biological characterization

**FIGURE 8:**
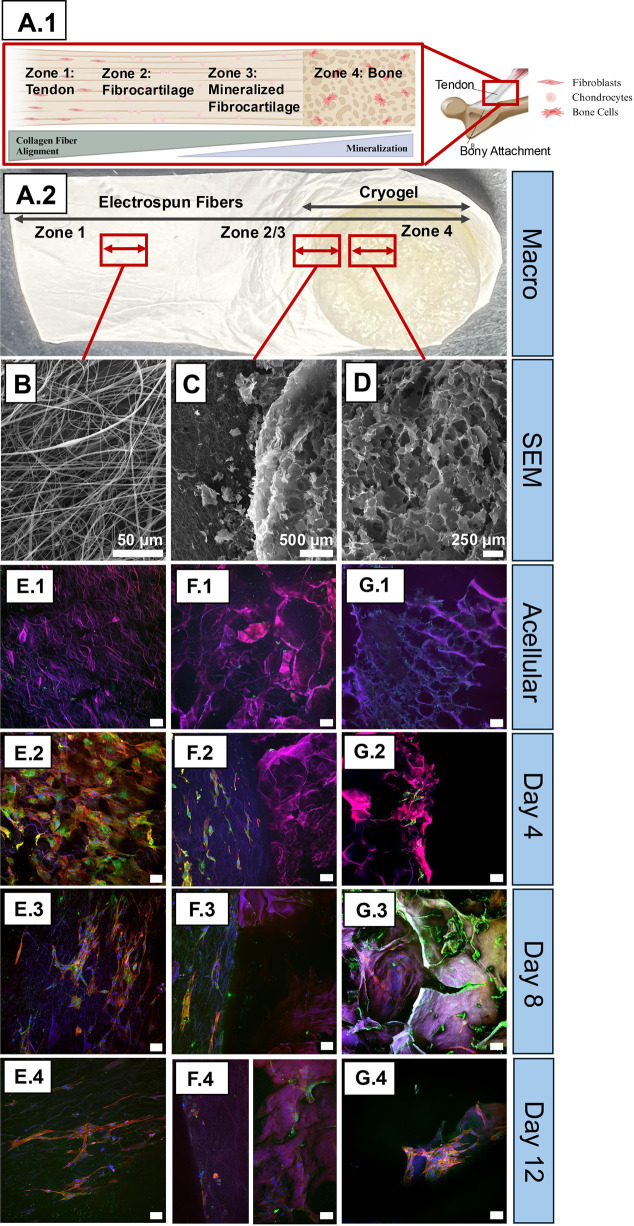
Combination ES and CG scaffold. **(A.1)** Graphic of the bone tendon interface including the four main zones, the gradient in collagen fiber alignment and mineralization, and the major cell populations found in the bone-tendon region. Image made in BioRender. **(A.2)** Macro scale image of the combined ES and CG scaffold. **(B–D)** Representative SEM images of three zones of the combined scaffold (zones 1, 2, and 3) at ×3000 (ES) and x200 (CG). **(E–G)** Confocal images of the three scaffold zones, with **(E.1, F.1, G.1)** showing autofluorescence of scaffolds without any cells seeded. The remaining confocal images **(E.3–G.4)** show representative images of the three scaffold zones at the timepoints: days 4, 8, and 12. Focal adhesions of ECM is marked by Anti-vinculin antibody (green), Actin in ECM stained by Rhodamine phalloidin-TRITC (red), and cell nuclei is stained by DAPI (blue).

For the cell study (Figure 8), nine controls of each final scaffold group (6% 50:50 PHB:SF ES and 6.5% SF 3–3 CG) were seeded with tenocytes and osteoblasts, respectively, and nine combined scaffolds were seeded with both tenocytes and osteoblasts. Figure 8A1, 2 depict a representative diagram and the macro view of the combined ES and CG scaffolds, highlighting the four zones in native tissue and the three zones targeted in this study. Figures 8B–D display the microscale view of the primary components of the combined scaffold via SEM, namely, the ES region, interface (ES + CG) region, and CG region. Note that both ES and CG scaffolds autofluoresced due to SF content (Semicheva et al., 2024); therefore, confocal images of scaffolds with no cells were included to demonstrate acellular controls (Figures 8E1–G1). Figures 8E2–G.4 shows cellularized scaffolds at three different timepoints (days 4, 8 and 12). Overall, the tendon cells were visually spread out over the ES scaffolds, as seen by the red actin fibers stretched in the direction of the ES fibers. Further, tendon cells on the ES scaffold were oriented along the fiber’s direction, as seen in Figure 8F2. In comparison, there were fewer cells on the CG portion, and they were less spread out/attached to the scaffold. There also appeared to be fewer cells on the ES scaffolds over longer periods of culture; day 4 ES scaffolds displayed more cells compared to days 8 and 12. When the ES and CG scaffolds were combined, the interfacial region, or where the ES and CG scaffolds integrate (Figures 8F2–F4), featured more cells on the ES portion; by day 12, the number of cells on the ES portion visually decreased. Note that Figure F.4 contains the ES and CG portions of the combined scaffold image separately due to the large size of the z stack being incompatible with acquiring a single image. Higher magnification images of the cryogel were also obtained to get a closer look at morphology and reduce background effects (Supplementary Figure S1).

**FIGURE 9:**
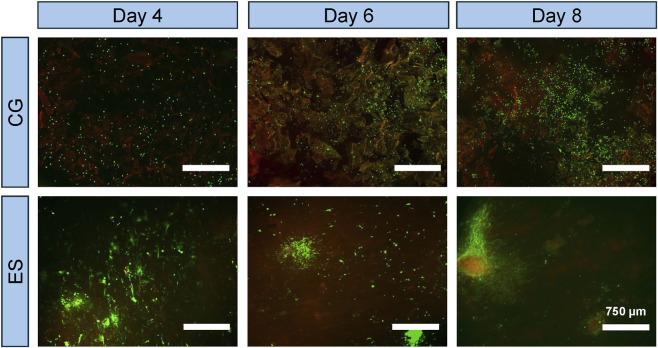
Live dead analysis on selected CG and ES samples at 4, 6, and 8 days, images taken at 4x.

Qualitative assessment via Live/Dead staining (Figure 9) demonstrated robust cell viability on the scaffold surface and a widespread distribution of viable cells throughout both the CG and ES scaffolds at all evaluated timepoints. By day 8, a high density of calcein-AM positive (live) cells with well-spread morphology was observed, indicating strong cell adhesion. In contrast, ethidium homodimer-1 (dead) signal was minimal to negligible across all samples.

## 4 Discussion

This study aimed to develop a proof-of-concept composite scaffold that combines PHB-SF electrospun mats and SF cryogels to mirror the mechanical, structural, and biochemical gradients found at the bone-tendon enthesis. The findings quantified the morphology (fiber diameter, fiber alignment, pore size), and tensile/compressive properties, which led to identifying optimal scaffolds to be used for combined scaffold development: i) % polymer and PHB:SF ratio for ES and ii) % SF and sonication mode for CG. The combined scaffold containing the optimal ES and CG scaffold components were then analyzed for cytocompatibility using tendon and bone cells. Further, the composite construct fabrication demonstrated the feasibility of engineering a seamless hard-soft bone-tendon interface.

It was generally observed that the addition of more PHB made samples brittle, and this was especially evident with the 100:0 samples; we hypothesize that this is due to PHB’s high crystallinity (Sarıkaya and Gümüşderelioğlu, 2021). When initially constructing a range of ES scaffolds for analysis, particular attention was given to analyze orientation (anisotropy) (Figure 1B) and fiber diameter (Figure 2B), both of which are critical for tendon applications. The high anisotropy values in ES samples with more PHB could be explained by PHB’s higher viscosity which was observed while making ES solutions. This resulted in less whipping action by the polymerous jet when increased PHB is incorporated into the ES mixture. In addition, it was noted that the addition of more PHB to SF resulted in a more stabilized Taylor cone during the ES process, which may have also contributed to a more uniform buildup of fibers on the collector (Moreir et al., 2024). Also, as total % polymer increased for ES scaffolds, there was a trend towards increased diameter. The slight significance in the interaction term (effect of % polymer and ratio PHB:SF) came from the 8% PHB:SF comparisons; interestingly, the fiber diameter decreased from 75:25 to 50:50 for the 8% samples but increased again in the 25:75 blend, making the trend different from four% to 6% samples. This was most likely due to the high ratio of SF lowering sample viscosity and the solvent not completely evaporating before the fibers reached the collector.

As the alignment for scaffolds was determined to be a crucial parameter of interest in developing tendon scaffolds, anisotropy ratio was used to quantify scaffold fiber alignment for all samples (Figure 2B). The maximum anisotropy achieved in this study was about 0.5; further investigations of RPM should be completed to optimize alignment.

After assessing fiber characteristics, tensile testing provides critical insight into the mechanical performance of ES scaffolds designed for tendon repair. The greater variability in elastic modulus (Figure 2C) with increased SF content is most likely related to the corresponding decreased anisotropy, as previously published papers have noted aligned fibers result in improved mechanical properties (Baker and Mauck, 2007; Robinson et al., 2021). In addition to increased variability in elastic modulus, there was an increasing trend in the average elastic modulus as the SF content increased, although not significant in this analysis (p > 0.05). Therefore, a greater SF composition makes ES scaffolds more resistant to elastic deformation. There are reports suggesting SF has a larger elastic moduli than PHB (Wang et al., 2004; Yeo et al., 2018); however, it must be noted that processing and stocks for electrospinning can highly impact mechanical properties. In this study, SF was extracted in house and although the process was done carefully with strict adherence to the protocol, there can be variability from stock to stock. PHB, on the other hand, was purchased. This could explain why there is a larger spread of error bars in elastic modulus data as SF content increased (Figure 2C). The optimal ES scaffolds were identified as the 6% 50:50 ES due to its relatively high and precise elastic modulus readings, with both fiber diameter in the micron-range which is well-suited for tendon applications (El Khatib et al., 2020; Gao et al., 2024), as well as consistent fiber morphology (no beading).

To determine which % SF and sonication mode produced the optimal CG for bone-tendon scaffold applications, pore diameter, swell testing, and compression elastic modulus were considered. It should first be noted that a sonication amplification mode of 80% was chosen in reference to previous work (Samal et al., 2013). The pore diameters for the 7.0% and 7.5% SF CG did not exhibit any significant differences among the pulsed settings (Figure 5A). While limited takeaways can be gleaned from the swell testing, the significant comparison between NP vs. three to three, the two extreme modes, in both 6.5% and 7%, suggest better swelling capability with three to three scaffolds at 24 h. This trend was also observed for the mechanical results, where there was only a significant difference between 7.0% NP and 3-3. Therefore, seven% and 7.5% CGs possessed similar properties, suggesting that once the % polymer exceeds a specific value, scaffold differences are negligible (Figure 5). The 6% three to three scaffolds featured most pore diameters in the 150+ micron range with a few smaller pores. Larger pores in the 200-micron range have been shown to be favorable for cell infiltration while smaller pores support cell attachment (Mukasheva et al., 2024). In addition, this scaffold featured a large elastic modulus and swelling capability, therefore it was chosen as the optimal CG for the combined construct.

Note that the 7.5% SF CGs appeared slightly misshapen with a jagged cross section, which is likely due to the high polymer concentration (Figure 4A). Specifically, the CGs fabricated from high polymer concentration (7 or 7.5%) were more likely to form gels that hardened prior to freezing. On the other hand, samples fabricated from very low SF %, specifically 5.5% SF, did not undergo the appropriate phase transformation from aqueous SF to a porous structure, leading to gels with a very high pore size distribution (heterogeneity) (Figure 5A) and variations in final cryogel structure (Figure 4C). This statement is further supported by swell testing where no results could be presented for 5.5% samples as these CGs broke into several pieces when placed in the PBS solution.

The primary reason for fabricating the CGs from SF was to keep the base material constant throughout the combined ES and CG scaffold. Fabrication by SF solution sonication in syringes create CGs with a wide range in pore sizes and characteristics (Figure 3B). Preliminary work for this study explored adjusting several parameters, such as sonication power, time, volume to sonicate, prior to additional % SF analysis and pulsing. Future studies should involve combining other polymers with SF, incorporating alternative crosslinking methods to enhance the reproducibility and mechanical properties of CG scaffold. For example, previous studies have investigated methacrylating the SF to support UV crosslinking (Mu et al., 2020).

**FIGURE 10:**
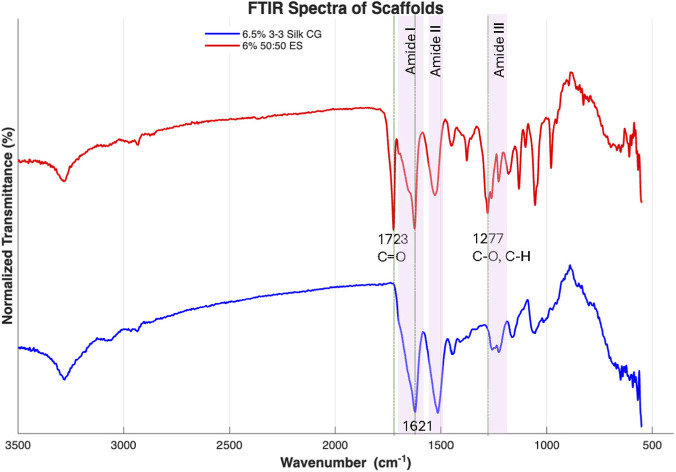
FTIR ATR spectra for 6% 50:50 ES scaffold (red) and 6.5% 3–3 CG scaffold (blue), with important peaks and regions labeled.

Figure 10 highlights a prominent Amide I peak centered at approximately 1,621 cm^-1^, which is consistent with a higher proportion of the β-sheet secondary structure in silk fibroin (Mao et al., 2021). This observation supports the crosslinked nature of both scaffold types and is expected given that both sonication (cryogels) and ethanol treatment for sterilization (for cryogels and electrospun scaffolds) are known to promote β-sheet formation and physical crosslinking in silk-based materials. A clear distinguishing feature of the electrospun scaffolds is the presence of the 1723 cm^-1^ peak, which represents the ester carbonyl of PHB, confirming successful incorporation of PHB within the composite fibers (Bhagowati et al., 2015).

Degradation results provide additional insight into scaffold stability and material-dependent behavior. Qualitative SEM analysis revealed visible degradation-related changes, including loss of pore structure and fiber integrity, in scaffolds incubated under protease conditions (Supplementary Figures S2, S4). In contrast, PBS-incubated groups exhibited higher percent initial mass values, suggesting that surface deposition or fluid uptake may partially mask hydrolytic degradation when assessed solely by mass change. SEM images support this interpretation, showing increasing surface deposition from Day 3 to Day 21, with evidence of deposition even at the earliest timepoint (Supplementary Figures S3, S5). Notably, this effect was substantially less pronounced in protease-treated. Lipase treated groups (Supplementary Figure S6) underwent a dramatic change in fiber architecture and also depicts some deposition. From the cryogel group, the absence of significant differences in mass across all groups and timepoints suggest these structures could be more resilient to degradation over treatment groups and timepoints suggests enhanced resistance to degradation, consistent with prior reports describing silk fibroin degradation on the order of months rather than weeks (Liu et al., 2015). PHB similarly exhibits degradation timescales on the order of months (Manavitehrani et al., 2016). Among ES formulations, 25:75 PHB:SF scaffolds demonstrated the greatest changes in mass over incubation. This behavior may be attributed to higher surface-area-to-volume ratio associated with smaller fiber diameters, increasing exposure of fiber surfaces to enzymatic and hydrolytic environments and thereby accelerating degradation-related effects.

Once the ideal ES and CG (6% 50:50% and 6.5% three to three pulse) scaffolds were chosen and characterized (Table 3 overviews summary of key morphological characteristics and their comparisons to native tissue), the combined construct could be made. The combined construct featured a CG with an ES layer spun on top (Figure 8A2). The sample was cut to show the three zones, and the intended variety in properties can be noted (Figures 8B–D). The place where the fibrous ES attached to the porous CG is the interfacial region, and both fibers and pores are pictured. A potential shortcoming of the model is that it features a sudden change in properties rather than a gradient, making it less like the native bone-tendon interface. Regardless, this model features changes in properties that can be used to approach understanding of cell interactions at the bone-tendon interface. Analysis of cell studies led to the conclusion that while both SF scaffold types can support cell growth (as supported by the presence of cells by day 12), ES scaffolds may be better-suited for long term cell viability (Figures 8E–G). ES scaffolds are known for their ECM-mimicking characteristics (Xue et al., 2019) and high cell attachment (Paşcu et al., 2013), where fiber alignment encourages directional cell growth (Baker and Mauck, 2007; Baker et al., 2012). Directional cell growth can be observed in Figures 8E2–2, and there appears to be less cell growth but more elongation with time. Figure 8E2 day 4 cells are rounder, while Figure 8E3, 4 feature long actin spindles along the direction of the ES fibers. CG cells were visibly less stretched and more clustered in groups, where the primary functions of pores in TE scaffolds is to support microenvironments within the scaffold and maintain close cell interactions (Mukasheva et al., 2024). Interestingly, it was more difficult to locate cells with all three stains visible in CGs as compared to ES scaffolds (e.g., Figure 8G2, E2). This is most likely due to limited cellular-stain penetration within the scaffolds. The combined construct had interesting results, as there seemed to be a decrease in stretched cells present on the ES side at day 12. The combined construct was more challenging to culture and image due to its irregular shape and, based on the rounded nature of the cells, it is possible that cells were disturbed prior to fixation. Another possibility is that the tenocyte cells matured quicker in the presence of the bone cell media, as it contains higher ascorbic acid, which can accelerate collagen synthesis (Wang et al., 1999). Future studies should investigate dynamic seeding options, methods to stabilize the combination scaffold during culture, and the optimization of cellular staining on SF CGs.

**TABLE 3:** Summary overview of key properties (elastic modulus, fiber diameter, and pore size) for human tendon and bone, and tissue engineered scaffolds fabricated in this study.

Type	Native tissue		TE scaffolds
TENDON	Flexor/Extensor	Larger tendons	Results
Elastic modulus	50–200 MPa (Schlecht, 2012)	1.5 GPa (Matson et al., 2012)	∼50 MPa
Fiber diameter	1–20 µm (Gomez-Florit et al., 2022)		∼1 µm

BONE	Cortical	Cancellous	Results
Elastic modulus	5–10 GPa (Hunt et al., 1998; Choi et al., 1990)	<5 GPa (Mu et al., 2020)	∼0.3–0.5 MPa
Pore size	​	300–600 µm (Jiao et al., 2023)	150–200 µm

The minimal detection of dead cells supports the high biocompatibility of the natural polymer matrices used in this study. Degradation products of both silk fibroin and PHB have been widely reported to be non-cytotoxic *in vitro* (Sapudom et al., 2023; Zhuikov et al., 2020). Furthermore, the absence of red fluorescence is likely influenced by the 3D nature of the assay; unlike in 2D culture systems where non-viable (dead) cells remain adhered the substrate, dead cells within porous scaffolds lose integrin-mediated adhesion and are frequently removed during the routine feeding and rinsing steps (Avnet et al., 2024). Additionally, cells exhibited more pronounced spreading on ES scaffolds compared to the CG samples and showed preferential alignment along fibers, consistent with contact guidance effects observed in fibrous scaffolds. This behavior was also evident in confocal imaging, further supporting the role of fiber architecture in directing cell morphology and organization. Several limitations of this study should be acknowledged. First, the mechanical properties of these scaffolds are lower than those of native bone-tendon tissues. This disparity is a well-recognized challenge in porous scaffold design, where a trade-off exists between mechanical stiffness and porosity (Stratton et al., 2016). While matching native stiffness is ideal for immediate load bearing applications, high porosity is critical during early stages of regeneration to facilitate cell infiltration, nutrient diffusion, and biological fixation. Literature suggests that scaffolds with lower elastic moduli may reduce stress shielding and promote cell adhesion, proliferation, and matrix deposition (Liu et al., 2025). Importantly, the mechanical properties of these constructs are expected to evolve over time as cells deposit ECM, which may contribute to increased stiffness and functional integration in future *in vitro* and *in vivo* studies (Baker and Mauck, 2007) There are also additives that could be explored to boost mechanical properties in these natural polymer scaffolds (Olevsky et al., 2023; Bakhtiari et al., 2025). Another limitation is the use of a simplified model containing only two different cell types, especially since the bone-tendon interface houses several different cell populations, three major ones being tenocytes, chondrocytes, and bone cells. Future work can involve more robust biological characterization of osteogenic/tenogenic markers as well as investigate the use of stem cells on these scaffolds. It would also be interesting to devise a method for mechanically stimulating these scaffolds for proper bone/tendon development, as this has been identified as key for enthesis formation (Killian, 2022). Notably, this work also does not consider the mechanical behavior at the interface of the combination construct, i.e., where the ES meets the CG. This is an important consideration in interface tissue engineering and should be assessed and optimized in future studies. Despite these limitations, this research adds to the foundation of work for tissue engineering the bone-tendon enthesis. The primary takeaways from this study included the development of a proof-of-concept composite scaffold that combines SF-PHB electrospun mats and SF cryogels to model the mechanical, structural, and biochemical gradients found at the bone-tendon enthesis. By characterizing each component individually via evaluating morphology (i.e., fiber diameter, fiber alignment, pore size), mechanical behavior (i.e., tensile and compressive properties), and cytocompatibility using relevant cell types, we were able to conclude that these scaffolds feature morphological characteristics that fall in range of native tissue values, mechanical behavior that approaches what is found in native tissue, and appropriate attachment sites for cells.

## 5 Conclusion

In summary, this study highlights the development of a tissue engineered construct created by combining two scaffolding techniques, cryogelation and electrospinning, to make a proof-of-concept replicate of the bone-tendon interface. By tuning parameters in both the ES (PHB:SF polymer ratio and %) and CG (% SF, sonication pulsing or no pulsing) scaffolds, a variety of characteristics were examined: anisotropy, fiber diameter, elastic modulus for ES and pore diameter, swell, elastic modulus for CG. From this physical characterization, an optimal scaffold group from each fabrication method (6% 50:50 for ES and 6.5% three to three pulse for CG) was chosen to fabricate the composite scaffold and for further cell analysis. The live/dead assay confirmed cell viability over time, and cell culture showed attachment in both the ES and CG, with more elongation on the ES portion and more cells present in the CG over time. Overall, SF polymer was shown to be a viable base material for fabricating bone-tendon composite scaffolds; however, additional investigation into the impact of dynamic culture and/or mechanical stimulation is required to identify if cell viability and crosstalk can be achieved and sustained for longer timepoints. Also, the mechanical properties at the combined scaffold interface (both in an acellular and cellularized form) should be investigated, as well as the impact of mineral addition on mechanical properties and supporting bone-like phenotypes. This study paves the way for future work focused on composite constructs for tissue interface engineering and provides a platform for future *in vivo* studies investigating regeneration at hard-soft tissue junctions, for which there is a high need in the field of tissue engineering.

## Data Availability

The raw data supporting the conclusions of this article will be made available by the authors, without undue reservation.
