# Supplementary material for: Silk cryogel and electrospun scaffold characterization for bone-tendon interface applications
Source: Front Bioeng Biotechnol. 2026 Mar 17;14:1685458. doi: 10.3389/fbioe.2026.1685458 (PMC13036107; doi:10.3389/fbioe.2026.1685458)
Supplement: Supplementary file 1 [file DataSheet1.pdf]

Polymer Concentration	PHB:SF Ratio	+Voltage (kV)	-Voltage (kV)	Flow Rate	Working Distance	RPM
4%	75:25	5.5	6.6	8 ml/hr	13	1500
4%	50:50	12.0	7.3-7.4	8/6 ml/hr	13	1500
4%	25:75	8.9	4.8	6 mlL/hr	12	1500
6%	75:25	7.0	6.0	8 ml/hr	12	1500
6%	50:50	5.8 - 6.6	6.6 - 6.7	8 ml/hr	13	1500
6%	25:75	7.2	6.6	8ml/hr	12	1500
8%	75:25	6.5	6.0	8 ml/hr	21	1500
8%	50:50	6.0	5.8	8 ml/hr	21	1500
8%	25:75	7.2	6.6	8 ml/hr	12	1500

Supplementary Table 1: Depicting electrospinning parameters used to develop each ES sample tested In this study.

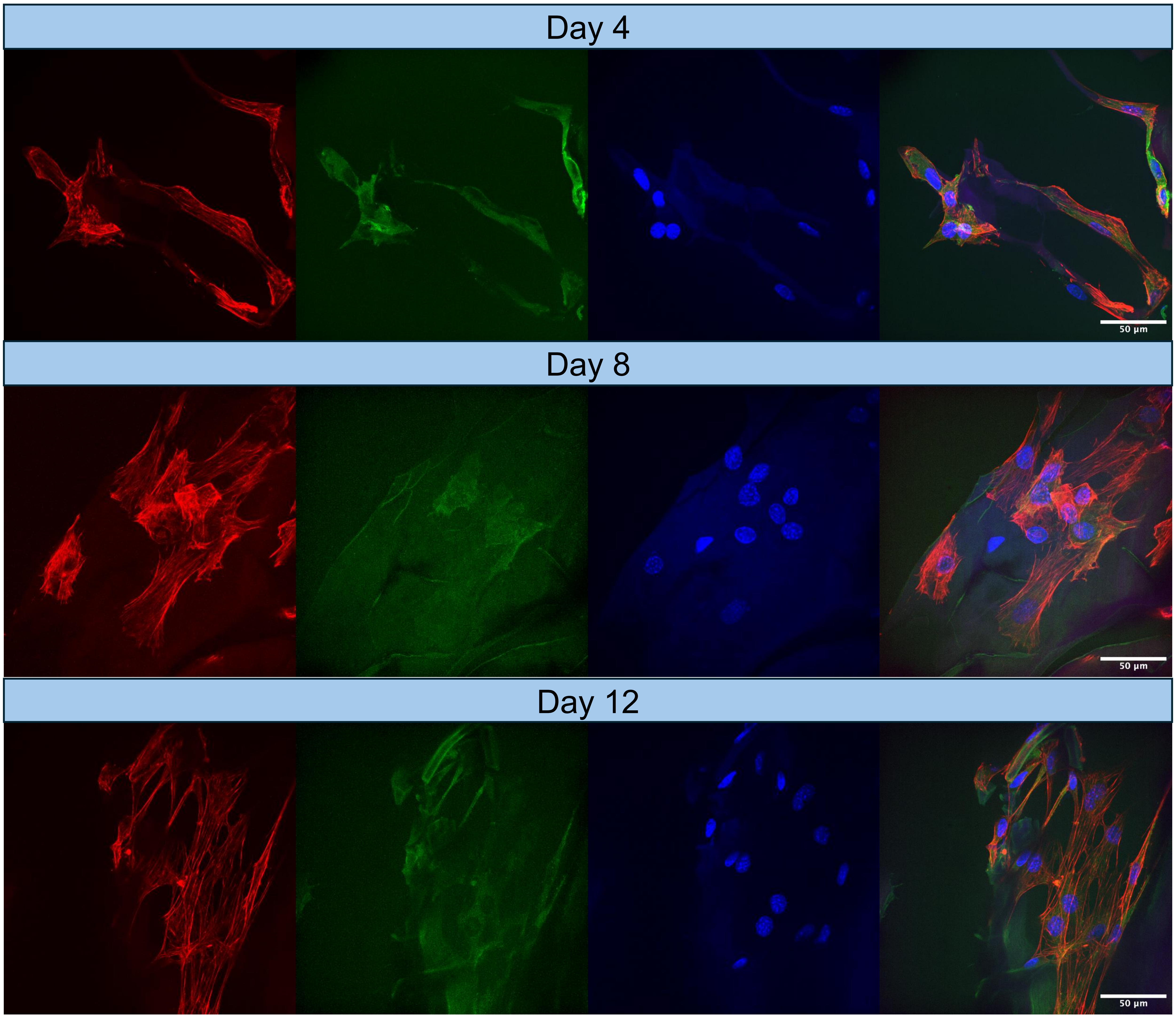

Supplementary Figure 1: Confocal images of the cryogel at 60x at days 4, 8, and 12. Focal adhesions of ECM is marked by Anti-vinculin antibody (green), Actin in ECM stained by Rhodamine phalloidin-TRITC (red), and cell nuclei is stained by DAPI (blue). These images were taken to obtain higher contrast / improved signal to noise ratio images from the cells on the cryogel and mitigate impacts of autofluorescence from the scaffold on cell morphology assessment. Scale bar shows 50 µm.

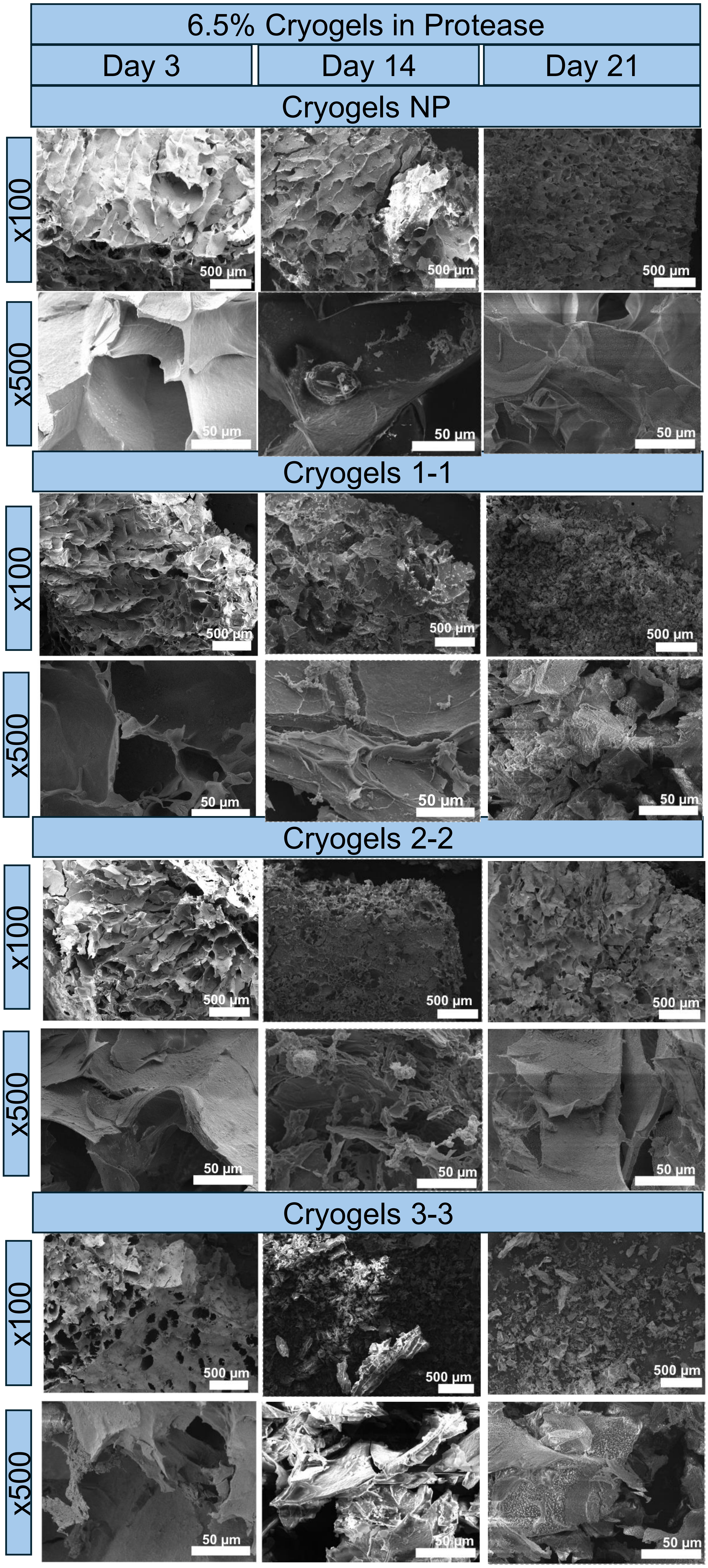

Supplementary Figure 2: SEM images at x100 and x500 detailing the pore structure and architecture as 6.5% cryogels were incubated in protease solution.

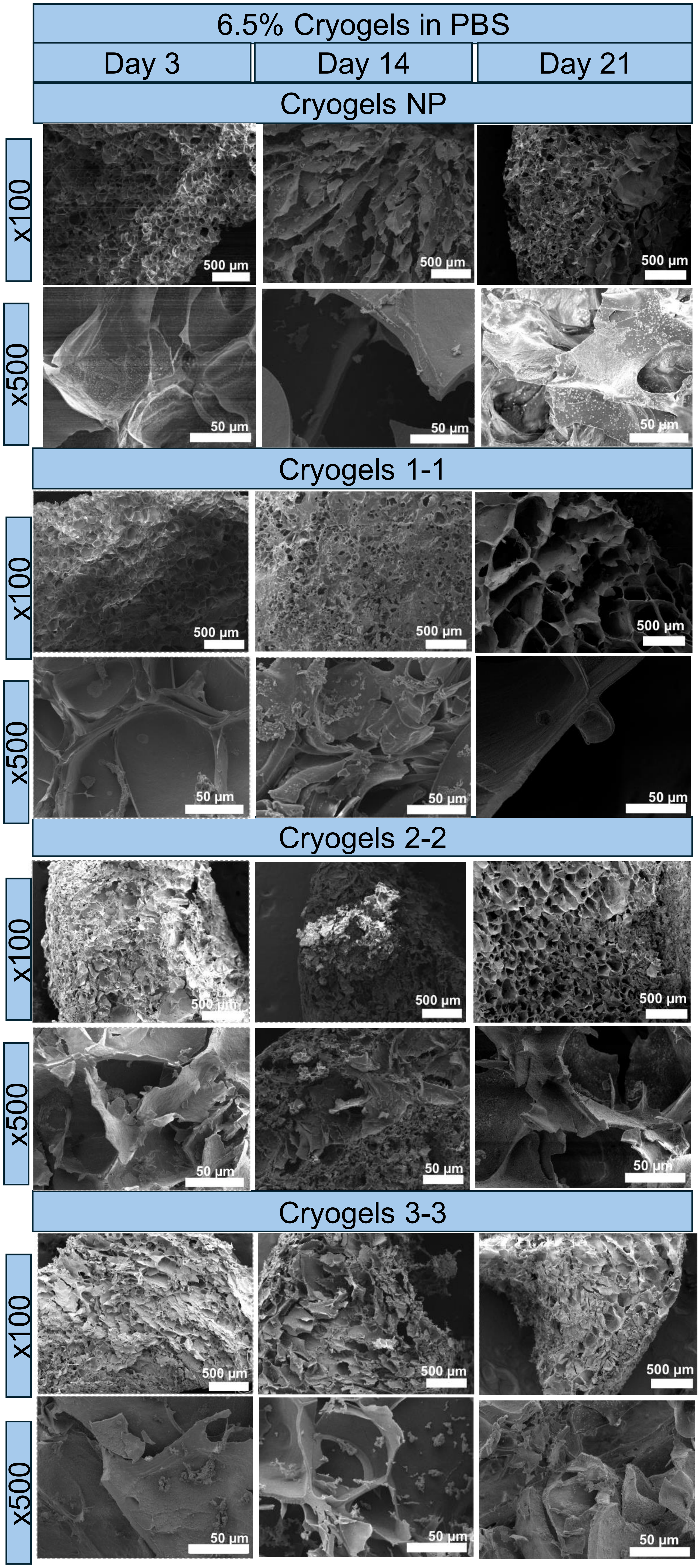

Supplementary Figure 3: SEM images at x100 and x500 detailing the pore structure and architecture as 6.5% cryogels were incubated in PBS solution.

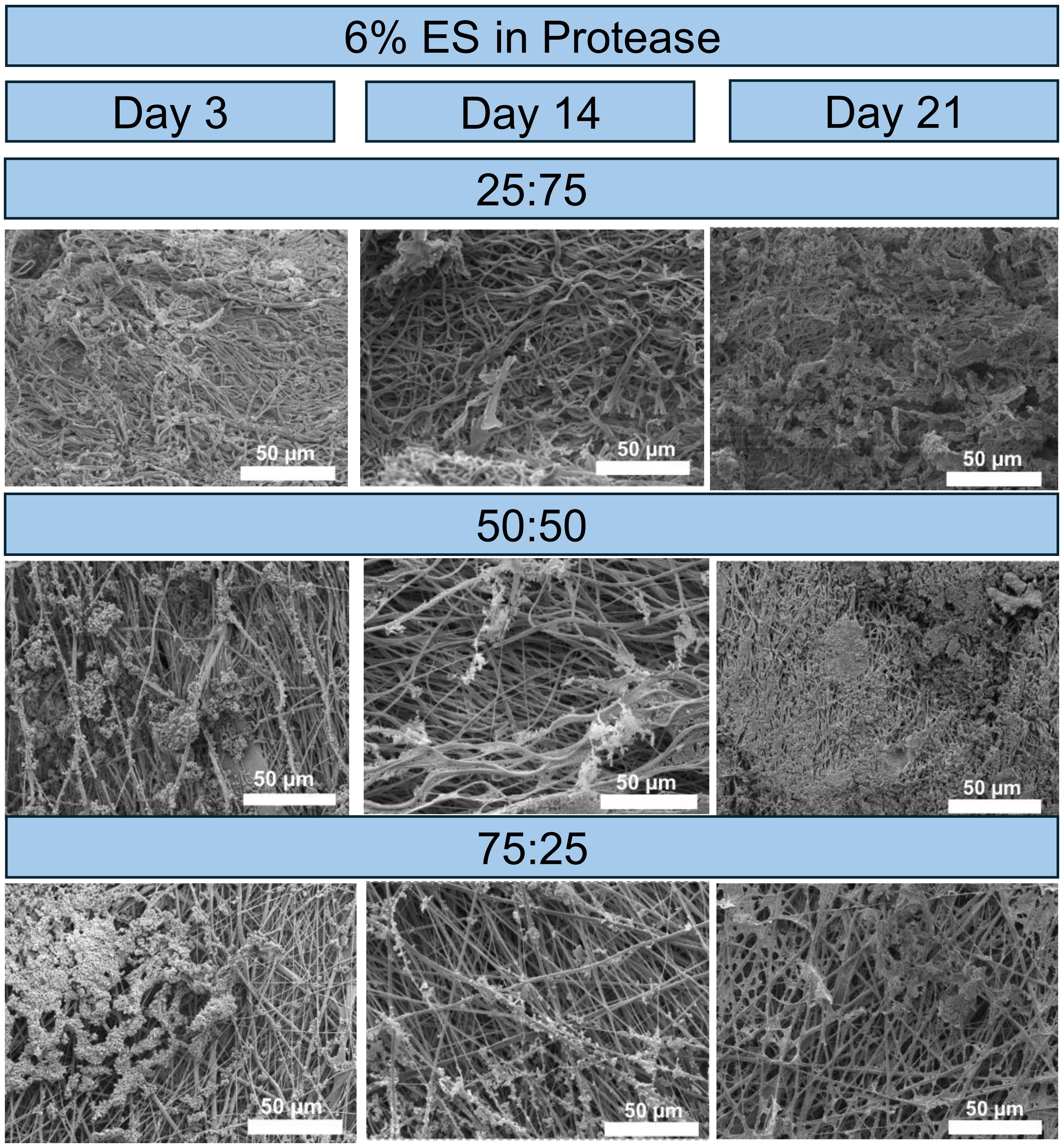

Supplementary Figure 4: SEM images at 100x and 500x detailing the fiber structure and architecture as 6% 50:50 ES were incubated in protease solution.

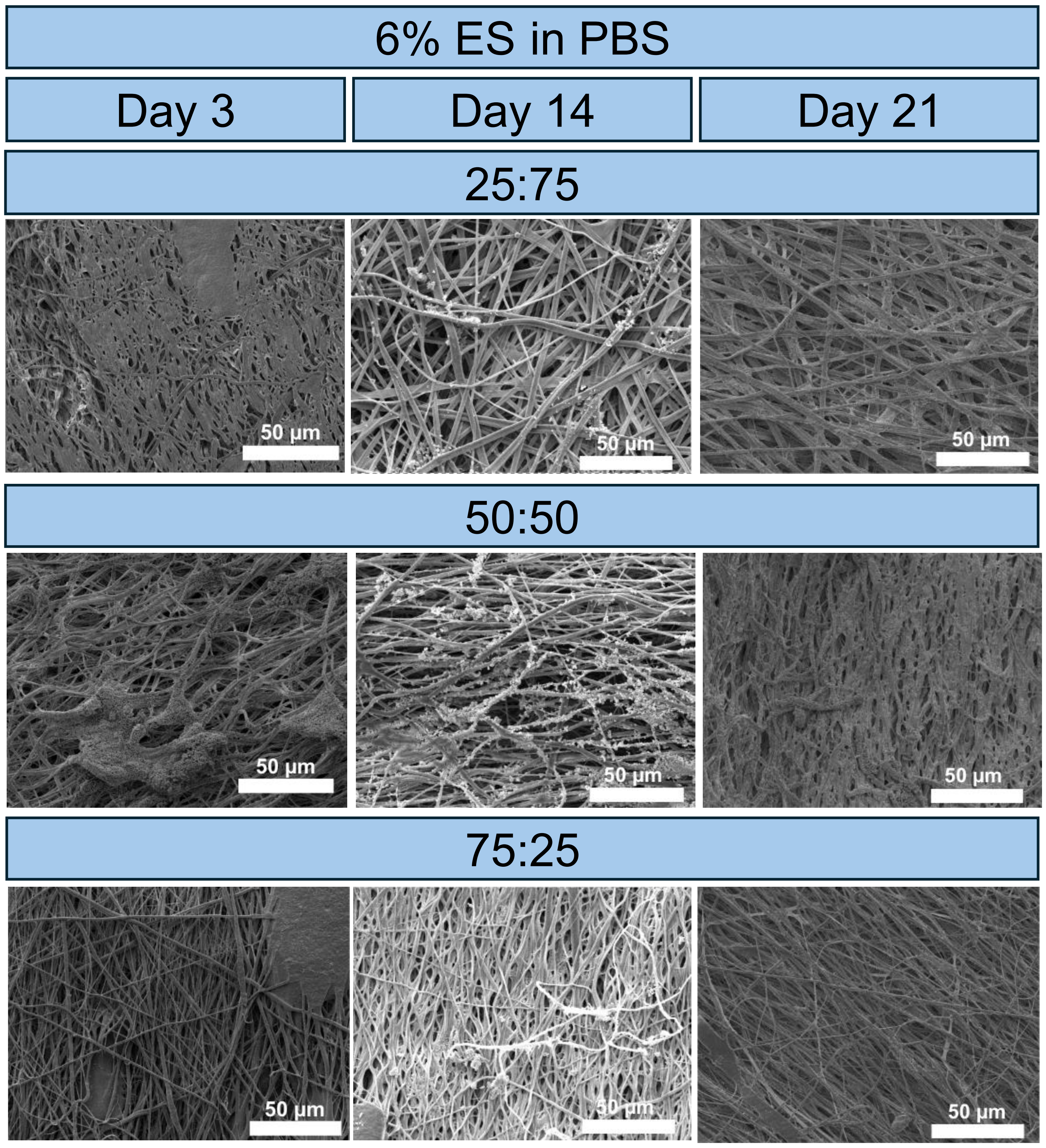

Supplementary Figure 5: SEM images at 100x and 500x detailing the fiber structure and architecture as 6% 50:50 ES were incubated in PBS solution.

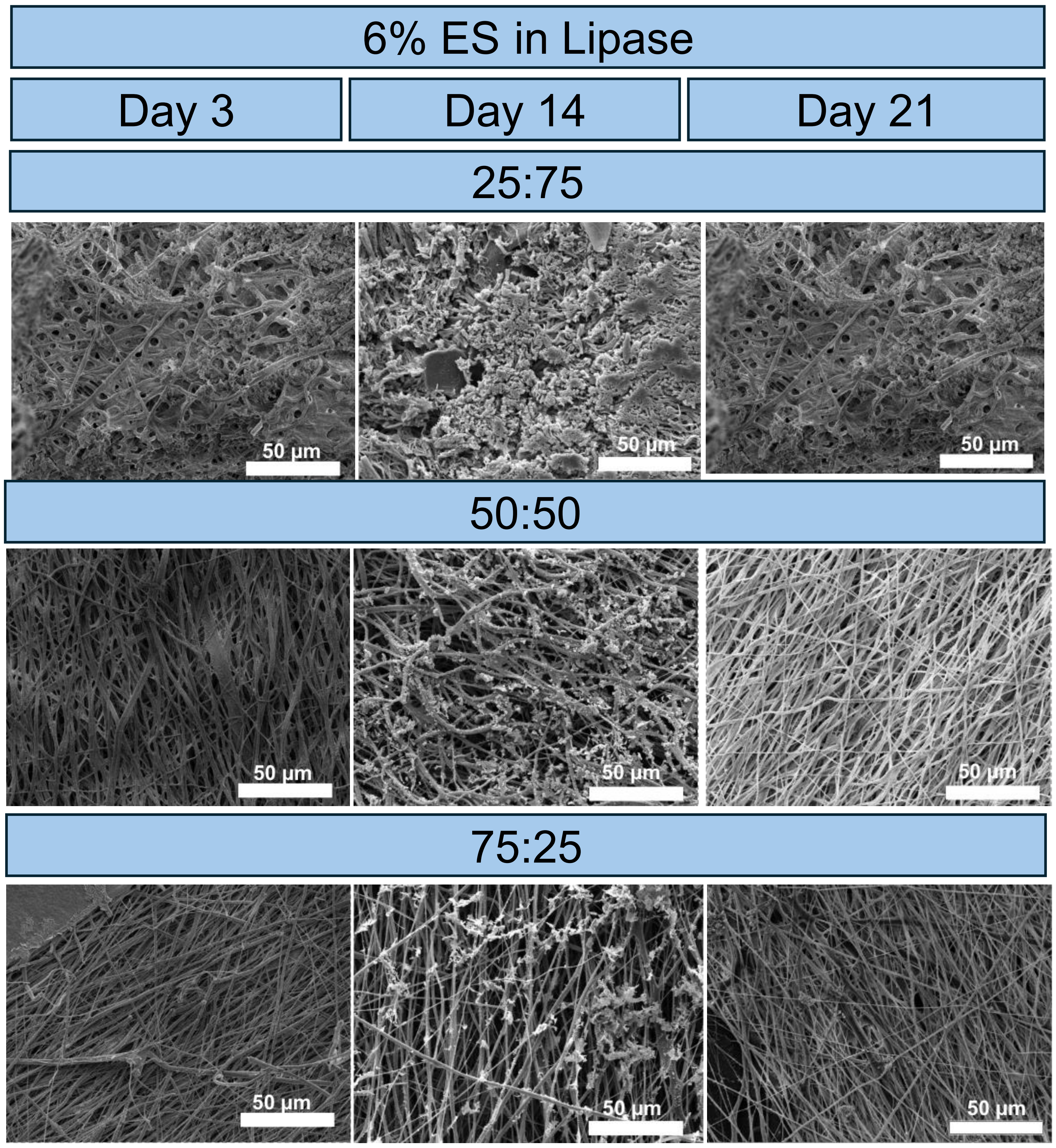

Supplementary Figure 6: SEM images at 100x and 500x detailing the fiber structure and architecture as 6% 50:50 ES were incubated in lipase solution.
